# UBL5 and Its Role in Viral Infections

**DOI:** 10.3390/v16121922

**Published:** 2024-12-16

**Authors:** Liancheng Xia, Yanhua He, Yifan Sui, Xijia Feng, Xijing Qian, Yangang Liu, Zhongtian Qi

**Affiliations:** 1 Key Laboratory of Biosafety Defense (Naval Medical University), Ministry of Education, Naval Medical University (Second Military Medical University), Shanghai 200433, China; xialiancheng@smmu.edu.cn (L.X.); yanhua0556@163.com (Y.H.); qianxijing@smmu.edu.cn (X.Q.); 2 College of Basic Medicine, Naval Medical University (Second Military Medical University), Shanghai 200433, China; alansui119@163.com (Y.S.); 15641980648@136.com (X.F.)

**Keywords:** ubiquitin-like protein 5, virus infection, pre-mRNA splicing, Fanconi anemia, unfolded protein response

## Abstract

Unlike other ubiquitin-like family members, UBL5 is structurally and functionally atypical, and a novel role in various biological processes and diseases has been discovered. UBL5 can stabilize the structure of the spliceosome, can promote post-transcriptional processing, and has been implicated in both DNA damage repair and protein unfolding reactions, as well as cellular mechanisms that are frequently exploited by viruses for their own proliferation during viral infections. In addition, UBL5 can inhibit viral infection by binding to the non-structural protein 3 of rice stripe virus and mediating its degradation. Therefore, UBL5 is an important link between viral infections and immunity, and its study will be beneficial for the prevention and treatment of viral infections in the future. However, a review of the current findings on the role of UBL5 in viral infection has not been undertaken. Therefore, in this review, we summarize the recent progress in understanding the functions of UBL5 and discuss its putative role in viral infections.

## 1. Introduction

Ubiquitin is a 76-amino acid small protein containing a carboxyl (C)-terminal diglycine (di-Gly) motif for target conjugation (ubiquitination), which is dependent on the sequential catalysis of ubiquitin-activating enzymes (E1), ubiquitin-conjugating enzymes (E2), and ubiquitin ligases (E3) [1]. The topology of ubiquitin chains, monomers, or polymers determines the fate of the substrate protein, guiding proteins to the proteasome for degradation, which is the best-known role of ubiquitin, or altering substrate activity by affecting metabolic stability, binding behavior, or localization [2].

Ubiquitin-like proteins (UBLs) are expressed in all eukaryotes and comprise a protein family that shares structural and evolutionary relationships with ubiquitin. These include SUMO, ISG15, UFM1, URM1, ATG8, ATG12, FAT10, RUB1/NEDD8, ESC2, FUBI, and UBL5 (Hub1) [3,4]. UBLs can be divided into two types based on their interactions with substrates: conjugated (type I UBLs) and unconjugated (type II UBLs) [5]. Type I UBLs, such as SUMO, ISG15, UFM1, URM1, ATG8, ATG12, FAT10, and RUB1/NEDD8, can be activated and conjugated to proteins, and their functions have been the subject of recent reviews [3,6,7,8]. For this reason, they are not covered in depth here. While ESC2, FUBI, and UBL5 always show structures similar to type I UBLs or have been observed in the context of E1-activating enzymes, E3 ligases, and Ub/Ubl proteases, they are considered type II UBLs, because they have not been observed to be conjugated with substrates [3].

Among the type II UBLs, UBL5, also known in the yeast *Saccharomyces cerevisiae* as homologous to ubiquitin 1 (Hub1), is the only UBL to lack the characteristic C-terminal di-Gly motif observed in other UBLs [9]. UBL5 is widely expressed in tissues and strongly conserved across phylogenies. Evidence of the role of UBL5 in stress response, obesity, cancer, and viral infection is continuously growing [2]. Here, we review recent progress in understanding the functions of UBL5 and discuss its putative role in viral infections.

## 2. Expression and Structure of the UBL5 Protein

UBL5, which was first identified during a screen for genes highly expressed in the human iris, is an evolutionarily conserved 73-amino-acid protein in the UBL family [10,11,12]. The porcine UBL5 gene has been mapped to the central portion of chromosome 2 (SSC2) at a position of 62.1 cM on the current USDA USMARC linkage map and 68.9 Mb on the Sscrofa 10.2 physical map [10]. Human UBL5 is currently located on chromosome 19p13.2 [13,14]. Variations in this gene have been reported as significantly associated with several quantitative metabolic syndrome-related traits [15,16]. However, one study of obese children showed no association between early onset obesity and UBL5 single-nucleotide polymorphisms (SNP) [17]. Although multiple alternatively spliced variants have been identified, the gene encodes the same 73-amino acid protein with a molecular weight of 8.5 kD and pI of 8.6 [18,19,20].

**Figure 1:**
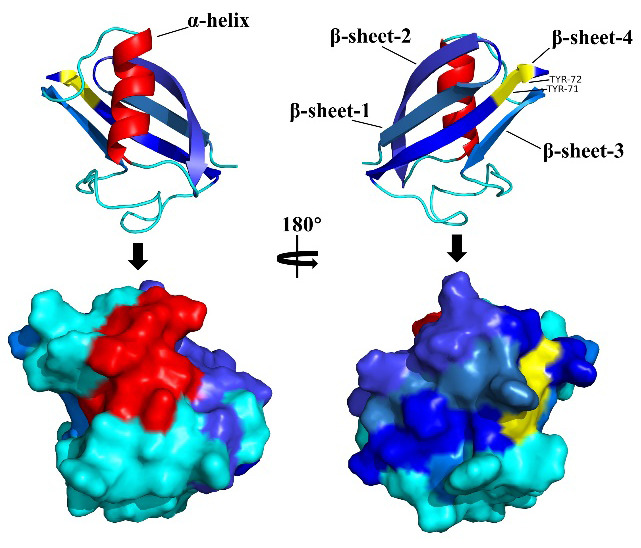
Three-dimensional structures of UBL5. The upper panel shows the structural ribbon representation of UBL5 (PDB:1P0R) and the lower panel shows the surface representation. α-helix is marked in red; β-sheets are marked in blue with different depths. C-terminal dityrosine (TYR-71 and TYR-72) motifs of UBL5 are highlighted in yellow.

The solution structures of UBL5 and Hub1 have been determined by nuclear magnetic resonance (NMR) spectroscopy and compared to that of ubiquitin [9,12]. Figure 1 shows the three-dimensional (3D) structure of UBL5. UBL5/Hub1, similarly to ubiquitin and other related proteins, adopts a ubiquitin-like β-scratch fold, consisting of four (UBL5) or five (Hub1) strands of antiparallel β-sheets surrounding an α-helix [9,12]. However, the critical surface residues of ubiquitin are not conserved in UBL5/Hub1, which may result in different functions [9]. The electrostatic surface of UBL5/Hub1 is highly charged, and no large hydrophobic patches are observed, which is different to that of ubiquitin [9]. The solution and crystal structure show that the secondary structure elements of UBL5 and Hub1 are β-β-α-β-β and β-β-α-β-β-3_10_-β, respectively, along the protein sequence [9,12]. Starting at the native N terminus, two antiparallel β-sheets are connected by a tight turn, followed by an α-helix which is connected to a third short sheet [12]. The final β-sheet of UBL5 is parallel to the second one and antiparallel to the short third one [12]. Notably, the unstructured tail C-terminal di-tyrosine residues, which are identical to those of the ubiquitin di-Gly GG motif and contained in the end of the final sheet, are involved in stabilizing the final β-sheet, but the residues are not exposed on the surface of the protein, and are not essential for UBL5/Hub1 binding with proteins [11,12]. NMR experiments have revealed that Hub1 has almost the same 3D structure as UBL5, except that it has a short β-sheet and α-helix before the final β-sheet (refer to the structure of UBL5) and the last residue in Hub1 is variable, which may be necessary for conjugation [9]. Furthermore, circular dichroism (CD), NMR, and X-ray analyses have revealed the interactions of Hub1 with the two N-terminal HINDs (HIND-I and HIND-II peptides) domain of the spliceosome protein, Snu66, which results in significant changes in several side chains on Hub1 [11]. Unlike the hydrophobic character and weak interaction between ubiquitin and its receptors, the Hub1–HIND interaction involves a strong salt bridge along with several hydrophobic contacts with high affinity, and its mode of interaction is different from the SUMO interacting with SUMO-interaction motifs (SIM) [11]. Through screening using yeast two-hybrid experiments, SIM and ferulic acid decarboxylase Fdc1a have also been identified as potential Hub1/UBL5/Hub1 interactors, suggesting that the binding site on UBL5 between HIND and SIM may be different [21]. Heteronuclear single quantum coherence (HSQC) NMR has further revealed that Hub1 binding induces the fold of HIND and increases the solubility of Snu66 [11].

## 3. UBL5 in Pre-mRNA Splicing

Splicing is an essential step in gene expression. Most eukaryotes convert precursor mRNA (pre-mRNA) to mRNA by splicing, at which point non-coding sequences (introns) are removed and coding sequences (exons) are joined [22,23]. In the process of splicing reaction, initially U1 snRNP will recognize the 5’-splice site of the intron. The intronic branch-site sequence will then bind to the U2 snRNP. Next, the pre-assembled U5 tri-snRNP is recruited and forms a pre-catalytic spliceosome. At each step of spliceosome assembly, the conformations of proteins and RNA molecules are rearranged and the spliceosome is activated with the help of several RNA helicases [24,25]. Accurate pre-mRNA splicing is absolutely necessary for proper gene expression; any error, either an error in single-nucleotide resolution or dysregulated splicing due to mutations in splicing factors, can lead to abnormal protein products, which ultimately leads to cell dysfunction and death [22,23,26,27].

Current research has shown that UBL5/Hub1 may influence the accuracy of pre-mRNA splicing by binding to U4/U6, U5 tri-snRNP, and the DEAD-box RNA helicase Prp5 in spliceosomes [11,28]. In the former pathway, UBL5/Hub1 binds to the Hub1 interaction domain (HIND) in Snu66 and Prp38, to promote the use of certain atypical 5′-splicing sites and alternative splicing of SRC1 pre-messenger RNA. The spliceosome lacking Hub1 cannot effectively utilize certain atypical 5′-splice sites and exhibits defects in alternative splicing of SRC1. This suggests that Hub1 plays a role in relaxing the specificity of spliceosomes, enabling them to handle diverse splicing sites effectively. The binding site is either at the N-terminus of SART1/Snu66 or at the C-terminal end of PRPF38A/Prp38, depending on the species [11]. Unlike the weak hydrophobic interactions between ubiquitin and ubiquitin receptors, UBL5/Hub1 interacts with HIND by forming a strong salt bridge between the conserved arginine of HIND and aspartate of UBL5/Hub1 [11,19]. The flexibility of this binding site suggests that Hub1 plays a broad role in spliceosomes and is not limited to specific binding partners. Although Hub1 is structurally similar to ubiquitin, it exhibits a unique non-covalent modification mode in terms of function. This correlation between structure and function reveals how Hub1 achieves flexibility in its function through different binding methods while maintaining its structure [11]. In another pathway, UBL5/Hub1 regulates pre-mRNA splicing by binding to the DEAD-box RNA helicase, Prp5 [28]. Prp5, an evolutionarily conserved RNA helicase in the DEAD-box protein family, forms a bridge between U1 and U2 snRNPs during pre-spliceosome formation. During this process, U1 and U2 binding is enhanced by SpPrp5 in the presence of ATP [29]. Karaduman et al. found that UBL5/Hub1 directly binds to and activates the ATPase activity of Prp5 through its second functional surface, thereby improving splicing efficiency. However, excessive activation can also lead to an increase in tolerance of suboptimal splicing sites and branching point sequences, which may cause splicing errors. The activation of splicing receptors mediated by UBL5/Hub1 induces the splicing of hidden introns in the PRP5 gene, which in turn inhibits Prp5 protein levels, thereby limiting excessive erroneous splicing [28].

Notably, yeast two-hybrid screens revealed that UBL5 interacts with all four members of the cell division control protein (CDC)-like kinase (CLK) [12,19,30]. The CLK family comprises an evolutionarily conserved family of serine/arginine-rich (SR) protein-modifying enzymes [31]. In most eukaryotes, selective splicing is regulated by SR proteins [32,33,34]. During this process, SR proteins are often phosphorylated by several SR protein kinases, which directly interact with pre-mRNA and stabilize the interactions between spliceosome components during the assembly process of spliceosomes [32,35]. The UBL5–CLK interaction may provide another pathway for the regulation of pre-mRNA splicing through SR protein-mediated regulation of alternative splicing. However, this hypothesis is yet to be proven.

## 4. UBL5 in the Fanconi Anemia (FA) Pathway

In addition to participating in pre-mRNA splicing, UBL5/Hub1 is involved in the FA pathway, a DNA damage response mechanism for repairing DNA strand interchain cross-links (ICLs) [36,37,38].

The FA DNA repair pathway mainly involves the repair of DNA strand interchain cross-links, which hinder replication and transcription, and is a particularly harmful type of DNA damage that requires complex repair mechanisms. During this process, multiple FA proteins synthesize FA complexes, catalyzing the mono-ubiquitination of FANCD2 and FANCI. Mono-ubiquitinated FANCD2 and FANCI subsequently relocate to DNA damage sites and interact with a series of DNA repair proteins to participate in DNA repair processes, such as homologous recombination (HR) repair and non-homologous end-joining [39]. Proteins in the FA pathway work synergistically with other DNA repair pathways such as nucleotide excision repair, mismatch repair, and HR, to identify and repair ICL damage. This process involves DNA unrolling, cutting damaged DNA fragments, synthesizing new DNA fragments, and reconnecting the DNA strands [40].

Oka et al. showed that UBL5 interacts specifically with the key component of the FA pathway, FANCI, thereby promoting the integrity of the FA pathway for ICL repair [37]. UBL5 binds to the aa261-280 region of FANCI. This enhances the overall function of the FA pathway and protects cells from the toxic effects of DNA strand cross-linking. UBL5 deficiency leads to a significant decrease in FANCI protein levels, which in turn affects the function of the FA pathway, manifesting as increased sensitivity to the DNA cross-linker, MMC, and impaired FANCD2 monoubiquitination [37].

When introducing point mutations in FANCI to block UBL5 binding, it has been reported that the FANCI Δ UBL5 mutant is unable to effectively form complexes with FANCD2. Due to the complete dependence of FANCI’s monoubiquitination on its binding to FANCD2, the monoubiquitination of the FANCI Δ UBL5 mutant is affected under both basal and DNA damage stress conditions. Interestingly, although the interaction between FANCI Δ UBL5 mutants and FANCD2 is impaired in cells, the FANCI Δ UBL5 mutant is still able to fully and effectively interact with FANCD2 in binding experiments using purified FANCI protein under strict conditions and recombinant FANCD2 in vitro. This suggests that UBL5 may play an intermediate role in promoting heterodimer formation of FANCI and FANCD2 within cells [37]. Through systematic mutation analysis, the D64A mutation in UBL5 weakens the binding of UBL5 to FANCI, but does not affect the function of UBL5 in pre-mRNA splicing, thus revealing the specific role of UBL5 in the FA pathway [37].

Recent studies have found that the interaction between FANCD2 and UBL5 is associated with the mitochondrial stress response and jointly participates in the regulation of the mitochondrial unfolded protein response (mtUPR) [36]. UBL5 may act as an activator of mtUPR, whereas FANCD2 exerts an inhibitory effect by jointly maintaining the transcriptional balance of the common fragile site (CFS) gene. Disruption of this balance may lead to the overexpression of CFS genes, increase chromosomal instability, and affect cell survival and adaptability [36].

## 5. UBL5 in mtUPR

MtUPR is a stress response to the abnormal accumulation of unfolded proteins during mitochondrial protein synthesis and transfer [41]. In this case, stress responses are rapidly activated to reduce the adverse effects caused by the abnormal accumulation of unfolded proteins by regulating the environment of protein folding through the regulation of translation and availability of chaperones and maintaining homeostasis of mitochondrial proteins. In a study by Benedetti et al. on nematodes under mitochondrial stress conditions, the accumulation of the UBL5-GFP fusion protein in the nucleus increased. This suggests that UBL5 may be involved in the cellular response to mitochondrial protein misfolding and exerts its role through nuclear localization [42]. The nuclear localization of UBL5 may be related to its role in mtUPRs. In the nucleus, UBL5 may be involved in regulating genes related to mitochondrial chaperone protein expression, thereby helping cells restore protein-folding balance within the mitochondria. The increase in nuclear localization may be an adaptive response of cells to mitochondrial stress that involves increasing the concentration of UBL5 in the nucleus and enhancing its ability to regulate the expression of related genes [42]. Moreover, inhibition of the UBL5 gene by RNA interference (RNAi) can inhibit the activation of mtUPR-labeled hsp-60tgfp and hsp-6tgfp and reduce the expression levels of endogenous mitochondrial chaperone-coding genes, hsp-60 and hsp-6. This further clarifies its role in coping with the mitochondrial stress response. Simultaneously, RNAi knockout of UBL-5 also affects the morphology of mitochondria and the assembly of multi-subunit mitochondrial complexes, indicating that UBL-5 also plays a role in combating physiological mitochondrial stress [42]. In addition, Cole et al. found that the complex of DVE1 and UBL5 undergoes nuclear redistribution during the activation of mtUPR, enhancing its binding ability to the mitochondrial chaperone gene promoter [43]. The enhancement of this binding ability may directly or indirectly affect the activity or localization of CLpP-1, thereby regulating the degradation of mitochondrial proteins through ubiquitination or other protein modification mechanisms. In a study on the stress response of *Caenorhabditis elegans*, Liu et al. found that in the intestinal mtUPR activated by nano-polystyrene particles, the expression of UBL5 and DVE1 increased and regulated the response of intestinal mtUPR through the Wnt and insulin signaling pathways [44]. Moreover, the expression of UBL5 and DVE-1 was regulated by upstream transcription factors, BAR-1 (β-catenin transcription factor) and DAF-16 (FOXO transcription factor), respectively [2,44].

## 6. UBL5 in ER Stress Response

With the continuous increase in research on the role of UBL5 in the mitochondrial unfolded protein response, it has been found that UBL5 plays an important role in cell death induced by the unfolded protein response protein kinase R-like endoplasmic reticulum kinase (UPR-PERK) arm and endoplasmic reticulum (ER) stress [45].

UBL5 plays an important role in the ER stress response. Under ER stress, the PERK pathway is activated, leading to decreased UBL5 stability. The UBL5 protein is rapidly degraded downstream of the UPR-PERK arm through the ubiquitin-independent proteasome system (UIPS), regulating the mtUPR signaling pathway and avoiding excessive or sustained mtUPR activation, leading to cell apoptosis.

## 7. UBL5 in Viral Infections

Once inside the host cell, viruses are unable to complete replication on their own. Most viral genomes are simple and poorly encoded, and must use host cellular mechanisms to replicate. This process involves replication, transcription, and post-transcriptional modifications. Host cells also have a range of mechanisms that respond to invading viral genomes; invasion and counterinvasion are complex and dynamic processes.

**Figure 2:**
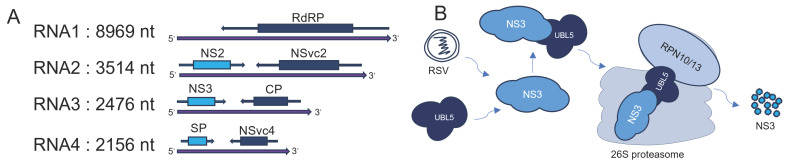
The role of UBL5 in the RSV infection process. This image shows the structure of the genome of RSV (**A**). RSV is a single-stranded RNA virus whose genome encodes four single-stranded RNAs. These encode the RNA-dependent RNA polymerase (RNA1), the NS2 and NSvc2 proteins (RNA2), the NS3 and nucleocapsid protein (RNA3), the SP and NSvc4 proteins (RNA4).The role of UBL5 in RSV infection (**B**). UBL5 binds to NS3 and is recognized by the RPN10/13 complex and then delivered for degradation in 26S proteasome.

Few studies have directly linked UBL5 to viral infections. However, UBL5 is likely to be an important host factor for viral infection. Rice stripe virus (RSV), a member of the genus Tenuivirus, can be transmitted by cyclic reproduction and causes many rice disease problems in China. The RSV is a single-stranded RNA virus with a genome comprising four single-stranded RNAs [46]. RNA1 encodes an RNA-dependent RNA polymerase (RdRP). RNA2–4 are double-sense RNAs. RNA2 encodes the NS2 and NSvc2 proteins of the virus, RNA3 encodes the NS3 and nucleocapsid proteins, and RNA4 encodes the SP and NSvc4 proteins [47] (Figure 2A).

In a study on the direct interaction of viruses with UBL5, it was found that UBL5 in *Nicotiana benthamiana* or rice could interact with the NS3 protein of RSV, a silencing repressor that regulates miRNA function by binding to microRNA processing complexes [48]. The NS3 protein can also bind to the sssiRNA and siRNA duplex to block the silencing signal propagation system [47]. This interaction can alter the cellular machinery to favor viral proliferation. The mode of action is for UBL5 to bind to the viral NS3 protein, which is then recognized by RPN10 and RPN13 and delivered by the RPN10/13 complex to the 26S proteasome for degradation [49] (Figure 2B).

In addition to direct research on UBL5 and the virus, there is much evidence suggesting that UBL5 plays an important role in viral infection. Influenza virus non-structural protein 1 (NS1) is a key protein in the resistance to host innate immunity and can promote viral infection by inhibiting dsRNA-induced host antiviral responses by targeting the RIG-I-mediated interferon pathway [50]. NS1 protein also binds to the stem-bulge structure of U6 snRNA during infection and inhibits the formation of U4/U6 complexes by binding to the 3′-end of U6 snRNA [51]. In another study, the Rev protein encoded by human immunodeficiency virus type 1 (HIV-1) interacted with its downstream proteins, and this interaction blocked the entry of U4, U5, and U6 snRNAs into the spliceosome to inhibit spliceosome formation [52]. This indicates that UBL5 may be important for the interaction between viral proteins and components of the spliceosome and viral infection.

Simultaneously, UBL5 has been identified as a viral infection-related protein using comprehensive proteomic analyses. Maintenance of infection by affecting host protein expression is a common mechanism of viral pathogenicity. Comprehensive proteomic analyses of SARS-CoV-2-infected cell lines expressing ACE2/TMPRSS2 revealed that the expression of numerous ubiquitin-related proteins, including UBL5, is downregulated. However, extensive transcriptomic results showed that, in the process of viral infection, expression of UBL5 did not show obvious changes except for HRV, which induced a mild decrease in UBL5 mRNA levels [53]. Notably, none of these studies have illustrated a positive role of UBL5 in viral infections. This may be due to the indispensable role of UBL5 for cell survival; CRISPR-mediated deletion of UBL5 potentially leads to cell death, and as a result, screening experiments have sequenced only surviving cells. Thus, more effective approaches are needed to determine the function of UBL5 in viral infections.

## 8. Conclusions

UBL5/Hub1 is a unique member of the type II UBL family that lacks a di-Gly at its C-terminus. Variations in these genes have been found to be significantly associated with several quantitative metabolic syndrome-related traits. The solution and crystal structure demonstrates that the secondary structure elements of UBL5 are β-β-α-β-β. As research progresses, the functions of UBL5 are continuously being explored, such as in pre-mRNA splicing, DNA repair, and the UFP response, some of which have been reported to be important in viral infection. A recent study has shown that it participates in viral infection by regulating the degradation of viral proteins. Conversely, during SARS-CoV-2 and HRV infection, the expression of UBL5 is downregulated. Collectively, the current literature reveals numerous functions of UBL5 in cellular life activities, predominantly associated with cellular stress responses and host functions essential for viral invasion of host cells. This indicates that UBL5 may act as a key factor in the regulation of viral infections. Nevertheless, studies on the precise function of UBL5 during viral infection remain rudimentary. This may be a promising avenue for future investigation in the study of viral infection and help to clarify the precise role of UBL5 in viral infections.
